# Medical students’ perceptions of integrating social media into a narrative medicine programme for 5th-year clerkship in Taiwan: a descriptive qualitative study

**DOI:** 10.1186/s12909-024-05255-y

**Published:** 2024-03-18

**Authors:** Yosika Septi Mauludina, Bao Lan Hoang, Tsai-Yu Wang, Chang-Chyi Jenq, Chi-Hsien Huang, Chien-Da Huang

**Affiliations:** 1 Chang Gung Medical Education Research Centre (CGMERC), Chang Gung Memorial Hospital, Linkou, Taiwan; 2 Department of Thoracic Medicine, Chang Gung Memorial Hospital, Chang Gung University College of Medicine, Taoyuan, Taiwan; 3 Department of Nephrology, Chang Gung Memorial Hospital, Chang Gung University College of Medicine, Taoyuan, Taiwan; 4 Department of Medical Education, Chang Gung Memorial Hospital, Linkou, Taiwan; 5 Department of Thoracic Medicine, Chang Gung Medical Education Research Centre (CGMERC), Chang Gung Memorial Hospital, 199 Tun Hua N. Rd, Taipei, Taiwan

**Keywords:** Medical students, Narrative medicine, Social media, Perception, Descriptive qualitative study

## Abstract

**Background:**

The growing demands in integrating digital pedagogies in learning (e.g., social media) contribute to disrupting many fields, including the medical humanities education. However, the strengths and barriers behind social media and medical humanities context are blurred and contradictive. We examined the perceptions of integrating social media – Facebook – into a narrative medicine (NM) programme for 5th -year clerkship in Taiwan.

**Methods:**

We used purposive sampling to recruit participants. Sixteen medical students (Female/Male: 7/9) participated in four group interviews. Semi-structured focus group interviews were conducted to explore students’ perceptions and experiences of the social media integrated into the NM programme. We analysed the data using a descriptive thematic analysis with a team-based approach. Data were managed and coded using ATLAS.ti version 9.0.

**Results:**

We identified six main themes: (1) Positive experiences of social media integration; (2) Negative experiences of social media integration; (3) Barriers on writing and sharing NM stories in social media; (4) Barriers on reading NM stories in social media; (5) Barriers on reacting contents in social media; (6) Suggestions for future improvement.

**Conclusions:**

The study revealed the strengths and barriers from medical students’ perceptions, when integrating social media into a NM programme. It is important to match students’ experiences, barriers, and perceptions towards learning. Understanding participants’ suggestions for future improvement are also crucial. With this knowledge, we might better develop the social media integration systems that achieve our desired outcomes based on the medical humanities education curricula.

**Supplementary Information:**

The online version contains supplementary material available at 10.1186/s12909-024-05255-y.

## Background

In the ever-evolving landscape of medical education and patient care, the intersection of social networking and medical humanities has raised complex questions and opportunities. The field of medical humanities represents a vital bridge between the clinical aspects of healthcare and the broader human experiences that define the patient-provider relationship [1, 2]. As medical education evolves to encompass a more holistic approach [3], the integration of social media platforms into medical humanities education has emerged as a promising avenue for enriching the learning experience [4]. The global outreach and ease of information sharing on social media platforms can enrich the humanities discourse [5, 6], fostering cross-cultural connections and facilitating valuable discussions [4].

However, this shift has introduced a nuanced interplay of effects on humanities pedagogic values [7]. While social networking may potentially contribute to a key approach to medical humanities education; enhancing reflection and collaboration in learning [7], the fundamentals in integrating social media into medical humanities context are blurred and contradictive [8]. For example, the medical humanities often emphasize narrative medicine (NM), which involves listening to and understanding patients’ stories [9]. While social networking platforms can serve as a stage for these narratives, they also have the capacity to foster superficial or unfinished storytelling [10], hindering the cultivation of essential skills among healthcare students and professionals, as they may not develop the ability to fully comprehend and appreciate the complexity of patient narratives. Meanwhile, there are challenges when integrating the tools into medical humanities education, including the superficial nature of many interactions [11], the potential for distraction, and the risk of creating echo chambers present challenges to the development of critical thinking and in-depth analysis in medical humanities education [12]. They are important issues for educators and students in preserving and enhancing the quality of medical humanities pedagogy in the digital age. As the rapid integration of technology and social media into healthcare and education, a thoughtful examination of the impact on the humanistic aspects of medical practice and education is needed,

According to social learning theory [13], the social aspect of learning is central, with interactions between individuals, peers, and the learning context shaping cognition and behavior, which includes knowledge exchange and cultural understanding. Integrating social networks into learning aligns with key elements of this theory, involving individual learners, peers, and situations that influence learning outcomes [14]. Furthermore, it encourages self-regulation in learning, prompting individuals to actively acquire and organize knowledge. Integrating social media such as Facebook has demonstrated benefits among students’ learning outcomes, including developing a sense of social learning and engagement within communities [14]. Furthermore, these platforms hold the potential to enhance students’ motivation through meaningful social connections [15], promote collaborative learning experiences [16], contribute to overall academic improvement by facilitating immediate and frequent feedback and sustained engagement [17].

Medical students use Facebook informally to enhance their learning and undergraduate lives and enable medical student students to create a supportive learning community amongst their peers [18]. A case study that involved the total 1749 medical student population found that 54.5% students were either using or open to using Facebook for educational purposes [19]. Notably, 27.7% of students using Facebook for educational reasons specifically utilized its ‘groups’ feature [19]. In addition, students typically using Facebook as part of their daily routines to engage in communication with their peers [20], imply that students see value in using Facebook as a means of communication and collaboration. This level of engagement may potentially enrich their learning experiences and foster a sense of community [20]. It reinforces the idea that integrating Facebook or similar social media platforms into educational contexts have the potential to support and enhance students’ educational journeys by aligning with their preferred modes of communication and interaction.

NM is an offspring of literature, medicine, and patient-centred care [21]. The content of NM in the medical humanities pedagogy is to prioritize the learning activities that promote reflective thought, writing through self-reflection, and narrative writing [2]. Thus medical professionals’ main focus is on the patient’s quality of care including multidimensional aspects such as biological, psychological, social, and emotional [22]. Therefore, medical students are required to develop their soft skills as future doctors have problem-solving skills, communication skills, and other skills that support their professional development [2]. In accordance with the constructivist theoretical framework for incorporating social media into medical education [23], this approach enables educators and students to interact and apply the learning process in more imaginative and innovative ways. These include fostering active engagement between learners and instructors, reducing the role of teachers as learners collaborate on group projects, fostering enhanced problem-solving skills, promoting self-directed learning, providing avenues for learners to engage in reflective thinking, and tailoring the learning environment to authentic contexts by utilizing problem-based and case-based materials [23, 24].

Despite the fact that social media has delivered considerable advantages and added value to educational initiatives within the medical humanities field, it has augmented conventional medical humanities education [4], improved communication and broadened accessibility [25], promoted collaborative teamwork [26], and increased students’ exposure to real-world practices and expert insights through enhanced interactivity [23]. Several studies conducted in Western countries have reported results on the medical student population [27, 28], highlighting the use of Facebook as a platform for sharing experiences in analyzing patients’ narrative stories [28]. These studies underscore the importance of raising awareness about the development of students’ professional identity and recognizing the role of social media in their lives, along with their responsibilities to future patients and the medical profession [27, 28]. To the best of our knowledge, there has been no exploration of the incorporation of social media, particularly in the context of NM, within the Asian medical humanities landscape. Consequently, our study is focused on assessing the effectiveness, barriers, and perceptions of integrating a social media -Facebook- as a social network into a NM program for 5th-year clerkship students in Taiwan.

## Methods

### Research context

The NM programme was conducted for a total of 12 weeks during the students’ clerkship in the Department of Internal Medicine, Chang Gung Memorial Hospital (CGMH) Linkou branch. A total of about 75 medical students in their 5th-year medical study joined the programme in each semester. The programme comprised a series of workshops, lecture classes, small group discussions, and story-telling. Additionally, we integrated the Facebook as a medium to facilitate students in the NM programme activities (Table 1). The rationale of this programme was to encourage teachers and trainees in fulfilling the aims of medical humanities education. It sought to advance medical humanities education by nurturing a sense of trust between mentors and trainees and by creating designated time for trainees to reflect on their clinical experiences. For integration of social media, the content of material posted by teachers on Facebook include students’ narrative stories, teachers’ responses, and humanities materials, et al. The students were invited into the Facebook Narrative Medicine group in a close system and encouraged to leave comments without compulsion. For the integration of the Facebook into the programme, the objective was to evaluate how medical students perceive the effectiveness of incorporating social media into medical humanities education. To facilitate this, a narrative approach was adopted, encouraging medical students to write about their day-to-day clinical interactions, challenges, and achievements with patients [2].

**Table 1:** The narrative medicine (NM) programme activities in 5th -year clerkship and additional programme in integrating social media into the programme practice

Programme activities	The details of the activity
Activity 1	The protocol for the narrative medicine (NM) program intervention (focussing on narrative writing) began with a typical lecture explaining the theory and introducing the narrative process. This activity was integrated as a one-hour session into the curriculum of Internal Medicine for Medical Clerkship in the first 2 weeks.
Activity 2	A NM workshop for clinical teachers. This workshop was held twice a year, and it covers the Brown Educational Guide to the Analysis of Narrative (BEGAN): a framework for enhancing the educational impact of faculty feedback on students’ reflective writing.
Activity 3	In this session during the clerkship of internal medicine for 12 weeks, medical students were invited to talk about clinical stories in their narrative writing assignments in different styles, such as storytelling or poetry-reading. This activity was designed to enhance humanism sensitivity among medical students by enabling them to recognize, interpret, and be moved into action by the problems of others. Through narrative writing, medical students could review their clerkship experiences, and they can rethink and reflect on the stories they gathered from patients. One of the objectives of the course is the opportunity for medical students to set their cognitive knowledge and skills in patient-physician communication into practice, thus facilitating a better understanding of patients and themselves. Professionalism teaching activity by nominal group technique will also be held.
Activity 4	This comprises a small group discussion among six to eight medical students and one clinical teacher facilitator in the final 2 weeks. In this one-hour activity, each student reads his/her narrative writing assignment, reflects on the experiences of their patient encounters and receives feedback from peers and the facilitator. The performance of each student was assessed through the adapted version of the Reflection Evaluation For Learners’ Enhanced Competencies Tool (REFLECT) guidelines.
Additional Activity	To implement the integration of social media – Facebook – into the NM programme, each week, the teacher and research members have posted some materials in the Facebook. The content of material posted by teachers on Facebook include students’ narrative stories, teachers’ responses, and humanities materials, et al. The students were invited into the Facebook Narrative Medicine group in a close system and encouraged to left comments and engage in the online interaction and discussion with the teachers and classmates without compulsion.

### Methodological orientation

The research in this study utilized a descriptive qualitative study approach, which is geared toward offering a straightforward depiction of a phenomenon [29], with the specific goal of informing the enhancement of programme interventions. The data was transcribed verbatim and analysed inductively.

### Sampling

We used a purposive sample technique to ensure a wide range of participants’ experiences, backgrounds, and attitudes [30]. The sample size was determined via theoretical saturation: we continued to recruit new participants until no new code was identified [31].

### Method of approach

We recruited participants from the School of Medicine at Chang Gung University, Taiwan. We focused on the 5th-year medical students as our study population since they had received the complete NM programme in their 5th clerkship.

We involved the secretary of the NM programme and the teachers in charge to inform them about the research plans and activities students publicly. The teachers in charge allowed research members to inform students about the integration of Facebook in the NM programme alongside specific rules for the Facebook activities. These rules included how to react, write and reflect, and comment on any content in the Facebook that were uploaded by research team members and teachers. During the research execution, teachers and research members posted content related to NM on the Facebook to encourage students’ interaction and discussion on the platform. The secretary of the NM programme in the Department of Internal Medicine and research team member (BLH) then announced publicly to the students about the recruitment of research participation. With students’ consent, allowed team members to contact them via email or phone in recruiting participants for the focus group interview and arranged their interview schedule. Informed consent was provided and small monetary rewards (NT dollars 250) was offered for participation. BLH was recruited the participants on a rolling basis until data saturation was reached or no new codes from the data analysis.

### Participants

Seventeen participants were initially recruited for the study. However, one participant had to withdraw due to scheduling conflicts, resulting in a total of sixteen medical students being interviewed. The students were organized into four focus group discussions based on their availability. Transcriptions of the interviews yielded four transcripts. All participants were of Taiwanese nationality, aged between 22 and 26 years old, and comprised of 7 females and 9 males.

### Setting and recruitment

This study was approved by the Institutional Review Board (Chang Gung Medical Foundation Institutional Review Board, CGMF-IRB) with certification of approval (202000437B0) for this Facebook activity involving students. The potential risks related to sharing stories or interacting online were mitigated under close Facebook group. Any strategies used to protect user privacy and confidentiality in the online environment were regulated and supervised by the CGMF-IRB. All methods were performed under the relevant guidelines and regulations.

### Researchers positioning

Our team consists of six members with diverse academic backgrounds and ethnicities. YSM, a female Indonesian research assistant, holds a Master of Science in physical therapy and public health. Proficient in interviews, data analysis, and qualitative software, she has made significant contributions to various qualitative studies. BLH, a male Vietnamese research assistant, holds a Master of Arts degree and is actively involved in linguistic and humanistic research, previously using qualitative study designs. CDH, CCJ, and TYW are male Taiwanese researchers with PhD and MD credentials who have actively conducted and contributed to research in the field of Medical Humanities.

### Data collection

Semi-structured focus group interviews were conducted to explore students’ perceptions and experiences of the social media integrated into the NM programme. We developed an interview outline based on our research questions and literature review related to this study (see appendix 1). Each interview lasted approximately 60 min. The interview was conducted in a quiet room in our medical center by BLH in English. Since some participants were more comfortable in Chinese answering the questions, one professional interpreter was involved in the interview to help the interviewer to get a better understanding and break the language barriers between the interviewer and participants. The relationship between the interviewers and participants was independent during the interviews with no power asymmetry. Negative effects on participants were minimized since the interviewer was not involved in any academic or professional activities with the participants. All interviews were audio-recorded, transcribed verbatim, and anonymized. All interviews were conducted in a single session, with no repeat interviews conducted. Participants were codified as STUDENT [number] respectively as their turn to speak at the beginning of the interview. Transcripts were codified as D [number] respectively as transcribed code in the analysis process.

### Data analysis

Data were managed and coded using the qualitative analysis software package ATLAS. ti version 9.0. Verbatim transcribing of the data in English was undertaken by YSM and BLH immediately after data collection. For data in Chinese, the translation process (from Mandarin to English was performed by a professional translator and carefully evaluated for translation accuracy by bilingual research members (CDH, CCJ, and TYW).

We used descriptive thematic analysis to analyze the data. Thematic analysis is a method for identifying, analyzing, and reporting patterns (themes) within data [32]. This kind of analysis is identifying and building up an analysis in a coherent matter (immersion) [32, 33]. The data were analyzed inductively. The data analysis began with YSM and BLH familiarizing themselves with the data through reading, reviewing, and re-reading the transcripts independently and noting down the meaning of each quote to generate initial codes, CDH and YSM was then subsequently collating codes to the potential themes, defined, and named the themes. The final process of the data analysis involved all research team members CDH, YSM, CCJ, BLH and TYW who reviewed, refined, and discuss themes and subthemes representing the whole data. Moreover, the team members actively considered the notion of data saturation. Any discrepancies were meticulously addressed through collaborative discussions until consensus was reached, ensuring the completion of the report [32]. Member checking was done through shared the final results representing participants’ quotes to participants via email to confirm the accuracy of data interpretation [34].

### Trustworthiness and rigor

The quality of our current study was assessed by core criteria identified by Lincoln and Guba [35]. The criteria include credibility, transferability, and confirmability. We used investigator triangulation in the whole process; involving one interviewer, BLH, two coders YSM and BLH, and all research members (YSM, BLH, CDH, TYW, CCJ, and CHH) were involved in the data analysis and writing of the report thus increasing the credibility and rigor of the whole analysis process. In this activity, codes and themes were continuously examined and discussed by the research team to ensure consistency. All research members were met frequently on an agreement basis, either online or face-to-face meetings for peer debriefing and progress reports. Lastly, we confirmed that our data analysis accurately reflected participants’ experience of the integration of social media into the programme through member checking by YSM via email with participants to ensure the final coding results were accurately reflected participants’ perception and experience in the integration social media – Facebook – into the NM programme.

## Results

Six main themes were derived from the data analysis: (1) Positive experiences of social media integration; (2) Negative experiences of social media integration; (3) Barriers on writing and sharing NM stories in social media; (4) Barriers on reading NM stories in social media; (5) Barriers on reacting contents in social media; (6) Suggestions for future improvement.

### Theme 1: positive experiences of social media integration

#### Subtheme 1–1 Facebook group facilitates student to streamlines thought-to-writing

*“…it (a Facebook) can record what we have said or else. Because sometimes you can’t figure out what you want to say (when writing up the Narrative story or experiences). …When faced with the challenge of expressing experiences, the platform allows for quick organization and clarity, ensuring our narratives are captured effectively.”* (S3-D4).

#### Subtheme I–2 enhancement of sharing others’ narrative articles by integration of Facebook

*“I think Facebook simplifies the process of discovering and sharing articles within our Narrative Medicine course, fostering a seamless exchange of insights.”* (S1-D1).

### Theme 2: negative experiences of social media integration

#### Subtheme 2–1 easily to get distraction

*“I think we get distracted easily, we want to watch or see other things online …we don’t spend time on study of medicine and prefer logging out or playing games, cause it more attractive for us”* (S1-D2).

*“…there are many things to distract our attention while online.”* (S1-D3).

#### Subtheme 2–2 Facebook cannot replace the face-to-face class

*“I don’t think that the group of narrative medicine can replace the real class. Yes, the real class could be more impressive, you can hear the experiences, emotions, and feelings of other classmates, and they can talk to you face-to-face. But, if you just look at the article or just read of some students’ articles, I don’t think it is fulfilled enough.”* (S4-D3).

*“I think if the social media is integrated with the online courses for biomedical knowledge, it is very good and yes, but for narrative medicine, I think the person-in-person experience by face-to-face class is more important.”* (S2-D1).

#### Subtheme 2–3 suboptimal for interacting with the NM content

*“…some senior doctors don’t have much time to use this Facebook to type their idea or what story they encounter. So, probably they shared a lot of the story when we have person-to-person interaction, they don’t share much their story or opinions on the Facebook*.” (S1-D1).

#### Subtheme 2–4 unmet students’ expectations

*“We have an online system called E-learning, and teachers post materials on that system and we can download it. So, when they informed us that we will use the Facebook, I expected that they will do something, like more personal interaction or ask each other opinions. But, it seems that the function is just the same as our E-learning system.”* (S2-D3).

*“I expected that whether there can be some skills or ways for us or to teach us on how to communicate with the patients.”* (S2-D4).

*“What I expected that teachers will share videos or tutorials to practice narrative medicine and how to communicate with patients.”* (S3-D4).

### Theme 3: barriers on writing and sharing NM stories in social media

#### Subtheme 3–1 unable to meet the tasks’ demands

*“Actually, we have some narrative stories during our practice in the hospital. But not every time we can write it into five hundred words of essay or paragraphs. Sometimes it is just two or three sentences and we shared it when we meet our colleagues on the way to the hospital or at night.”* (S2-D3).

*“…one of our classmates did not submit his homework of the narrative medicine story writing, because he said that he didn’t have any impressive or unforgettable moment related to the medical humanities practice.”* (S3-D3).

*“…people have a lot of feelings and ideas, but only a few people are able to change them into words.”* (S5-D3).

#### Subtheme 3–2 writing doubt deters platform sharing

*…if you think that your writing is not good enough, you would prefer not to post it in a Facebook.”* (S3-D3).

### Theme 4: barriers on reading the NM stories in social media

#### Subtheme 4–1 lack of time and supervision from teachers

*“Actually, because the teacher didn’t know whether we read it or not. So, I think I don’t need to read it.”* (Student added) “… *and we do not have much time to see it. Besides, I can learn it face-to-face. So, I didn’t take notes at all. …I didn’t absorb any knowledge from the article.”* (S1-D3).

*“It’s hard to finish or to read all articles, and I think there are even people older than us (who has busier schedule) that many of us don’t have time to finish all of the articles.”* (S1-D1).

#### Subtheme 4–2 doubt emerges when stories diverge from personal or observed experiences

*“Some stories may not attractive for me. I see some stories make me think that it is fake or not … yeah, because in my experience I didn’t see his or her experience like what they written up before even when I see my teacher in a clinical presentation, it made myself have a suspicious thought for these kinds of articles.”* (S3-D2).

### Theme 5: barriers on reacting contents in social media

#### Subtheme 5–1 personal factors

*“I feel shy to post …or leaving comments publicly.”* (S1, S5-D3).

*“…our classmate may not be very comfortable (if we react on their article) and we are embarrassed to leave some message.”* (S3-D4).

#### Subtheme 5–2 inter-personal factors

*“…because we were not to encouraged to share our opinion in front of the teacher…most of the time we just ask about teachers’ opinions and uncommon to share our opinion publicly or share what we learned with them. So, sometimes we are afraid of being criticized by others or just being afraid to say.”* (S1-D3) and *“…give us little more encouragement.”* (S4-D4).

#### Subtheme 5–3 cultural factors

*“I think culture issue is one thing that causes Asian students like us tend to shy and keep our opinion to ourselves.”* (S2-D3).

*“…we don’t comment much in the Facebook if we are not familiar or very interested with and even on the Facebook fan page that we are interested in we also may not leave any comment like that.”* (S1-D2).

#### Subtheme 5–4 technical factors

*“We use it as a media that teachers announce to us what time to go to the class, the classroom announcement, and how we can join the class.”* (S5-D3) and *“I only use it to check whether there is some information about the class.”* (S1-D1).

*“If someone posts something new in the narrative medicine group, but we view this group rarely, then the post will not show on our Facebook feeds, so we wouldn’t see it.”* (S1-D2) and *“…I don’t even know the article in there.”* (S1-D1).

### Theme 6: suggestions for future improvement

#### Subtheme 6–1 feedback and rewards from teachers

*“…Teachers want us to write down our experiences in the clinical practice, so it can be combined with the form of a literature contest. We can submit the articles and then we can review them with teachers. So, it’s like a competition and the winner can get some prizes …yeah like to encourage us, it can be a small incentive for students.”* (S1, S2-D1).

*“…we can draw a lucky prize or have a gift for the good comments or for students who post the most comments on the articles.”* (S1, S3-D2).

#### Subtheme 6–2 content improvement in social media platform

*“We follow many medical associate groups and they posted some medical knowledge in a very simple way for us to understand. So, I think it’s really important if you want us to have more attention to narrative medicine (through social media) I think more activities or some cooperation with some famous people would be more attractive.”* (S1-D2).

#### Subtheme 6–3 social media platform improvement

*“…someone who posts the contents can be hidden their name instead of show their name on Facebook. I think it would be helpful for us and people to post their experiences and opinions.”* (S3-D2).

*“…anonymous can maybe more stable for this class, at least post should be anonymous. So, when we leave some comments, we are not worried about being criticized*.” (S1-D3).

*“I think maybe they (teachers) can link to another kind of media, …yeah it is more attractive way like connect it with podcasts or some media that can let us share our idea in a more clearly.”* (S5-D1).

## Discussion

Integrating social media into a NM programme for 5-year clerkship medical students is a novel approach within the field of medical humanities education. Our findings reveal that this integration has both favorable and unfavorable dimensions, which are significantly shaped by the prevailing learning culture, values, attitudes towards popular culture, individual behavior, and personal choices.

When implementing the Facebook in a NM program, medical students recognize its role in facilitating their learning journey. However, they also acknowledge that it cannot serve as a complete substitute for in-person classes. In general, the medical students are accustomed to the university’s internal e-learning system, which enables easy access, utilization, and downloading of all course materials. When the social media is integrated with the online courses for biomedical knowledge, like e-learning system, it could be good as e-learning appears to be at least as effective as traditional instructor-led methods such as lectures [36]. However, NM fundamentally revolves around nurturing the humanistic aspects of medicine, which involve empathizing with and reflecting upon the emotions of others in the context of narratives [22]. Consequently, integrating social media without face-to-face interaction is perceived as less effective in this context [37], Moreover, the outcomes of NM heavily depend on the development of interpersonal relationships, the cultivation of empathy, and the ability to adopt different perspectives. Thus, face-to-face classes that encourage person-to-person interactions are preferred for NM programme activities over textual content on social media platforms. In addition, the current trend in social media consumption emphasizes platforms that employ audio-visual content, including recorded videos, live streams, creative visuals, and podcasts [38, 39]. It is essential for programme developers, educators, and facilitators to consider these preferences when integrating social media into the learning process for students.

Although Line has been the dominant social media platform in Taiwan since it overtook Facebook in 2018, Facebook is still widely used by approximately 90% of Taiwanese people [40]. While Facebook remains one of the most popular social media platforms, the broader culture of social media usage significantly influences our students’ choices and how they incorporate it into their daily lives. Our medical students commonly utilize Facebook for communication and accessing information related to their coursework and class schedules. Consequently, introducing the Facebook into the curriculum as a platform for idea sharing and discussions may not align with their preferences. However, considering the prevalence of other social media tools that have permeated popular culture, integrating these alternative platforms could potentially increase student engagement in the learning process. For instance, the American Academy of Physical Medicine & Rehabilitation hosts “Phyzforum,” a social network designed for sharing ideas, asking questions, and providing comments [41]. Another well-known discussion platform is SERMO, which serves as a valuable tool for healthcare professionals to connect with their peers, enabling them to exchange diverse experiences and strategies related to various medical conditions and an array of peer-to-peer subjects [42]. Our results indicate that it is essential to utilize current platforms that align with students’ requirements and inclinations to achieve favorable results.

Our research identified several factors that serve as barriers to the integration of social media into NM programs. These factors encompass personal, interpersonal, cultural, and technical aspects, and they significantly impact students’ engagement in the program. Our findings reveal that students’ online learning behaviors are greatly shaped by their personal values. One noteworthy observation is that students tend to withhold their ideas, particularly when they feel uncertain about their writing abilities. This reluctance to share ideas has noticeable repercussions on students’ motivation as active participants in online learning environments. Their lack of familiarity with publicly commenting and the desire for private spaces to provide feedback to their peers have led to reduced participation in the Facebook, resulting in limited interaction and a lack of relationship development with fellow students and colleagues. For cultural aspects, feelings of shyness, insecurity, and apprehension about receiving negative feedback from peers, specific to Taiwan, underscore the adverse effects of using social media in the learning process. We agree that students’ shyness, insecurity, and concern about receiving negative feedback from peers belong to students’ inner thoughts/expressions. However, Confucianist learning tradition is deeply ingrained in East Asian education [43]. Confucian culture holds considerable sway over various aspects of society and is notably influential in healthcare research and medical education in East Asian countries [44, 45]. The influence of Confucianism on learning styles in medical education warrants examination within the broader context of local cultural influences in East Asia. As a result, a majority of students recommend the incorporation of an anonymous feature within the learning platform when integrating social media into the NM program. This finding aligned with the potential factor associated with students’ focus on teaching online [46–48]. In addition, anonymity was important in the pedagogical implication that affects posting behavior factors, such as online privacy concern [46], self-consciousness [49], fear of negative evaluation [50], trust in the virtual community [51], and perceived psychological safety [49], and self-efficacy [49]. This finding implies that allowing for anonymous posts in online discussion boards could potentially enhance student engagement by creating a psychologically secure learning environment that mitigates the influence of self-consciousness and the fear of negative assessment on posting activities.

Evaluating and aligning students’ learning requirements and expectations with the available faculty resources and institutional support is crucial for enhancing future programs. The outcomes of this study clearly emphasize the need for an assessment of integrating social media technologies into medical humanities education, particularly in the context of the NM program. This assessment should take into account the preferences of users. To better cater to the needs of medical students in the integration of social media into the medical humanities learning context, improvements in content quality, adoption of current platforms, and program evaluation must be prioritized.

### Limitation

This study is subject to several limitations. Firstly, the study’s participants were confined to 5th-year medical students within a single site. Future research should aim to encompass a more extensive range of medical centers to augment the diversity of participant backgrounds, perspectives, and attitudes towards learning. Secondly, this study exclusively focused on integrating the Facebook into the NM programme. Further research is warranted to delve deeper into the integration of various social media platforms into the context of medical humanities education to gain a more comprehensive understanding.

## Conclusion

The integration of social media into the realm of education is increasingly gaining popularity. However, in Taiwan, using the Facebook as a means to discuss and interact within the context of the NM programme was unfamiliar. This is primarily because the prevailing culture among our learners typically uses Facebook as a medium for accessing course materials and programme-related information. Social media, in general, comes with both positive and negative aspects when applied to the learning process. When incorporating social media into educational practices, it is essential to have a firm grasp of the platforms that participants are currently using. Aligning these tools with students’ values, cultural backgrounds, and learning attitudes becomes a critical consideration in the integration of social media into medical education practices. Furthermore, it is crucial to conduct in-depth research to understand the perspectives of faculty members and educators regarding the utilization of social media in the learning process. This exploration is vital for the development of an enhanced e-learning system that can effectively deliver outcomes in alignment with the curricula within medical humanities education.

### Electronic supplementary material

Below is the link to the electronic supplementary material.

Supplementary Material 1

## Data Availability

No datasets were generated or analysed during the current study.

## Abbreviations

AMA: American Medical Association
CGMF-IRB: Chang Gung Medical Foundation Institutional Review Board
NM: Narrative Medicine
REFLECT: Reflection Evaluation For Learners’ Enhanced Competencies Tool
