# Supplementary material for: Medical students’ perceptions of integrating social media into a narrative medicine programme for 5th-year clerkship in Taiwan: a descriptive qualitative study
Source: BMC Med Educ. 2024 Mar 18;24:300. doi: 10.1186/s12909-024-05255-y (PMC10949758; doi:10.1186/s12909-024-05255-y)
Supplement: Supplementary file 1 — Supplementary Material 1 [file 12909_2024_5255_MOESM1_ESM.docx]

**Appendix 1*.* Semi-structured interview questions for medical students who use a social media ( Facebook), integration into the narrative medicine (NM) programme**

| **Opening Question** | What is your overal experience of the programme with the integration of the social media to the narrative medicine (NM)? Can you give an example to specify your saying? |
| --- | --- |
| **Introductory Question** | What is (are) the purpose(s) of the integration of the social media to the programme in your opinion? Could you give an example to specify your saying? |
| **Transition Question** | What do you wish to benefit from the integration of the social media to the progammme? What hinders your learning in this new approach? And what helps your learning in this new approach? Could you give some examples? |
|  | Have you ever applied what you have learned from NM programme into clinical settings? Can you tell me the situation? Could you give an example to help me understand? |
| **Key questions** | Could you tell me your understanding of the importance of social media in medical education? And how can the social media improve the course in your opinion? |
|  | Could you tell me the difference between you, those who posted and commented the most and those who posted and commented the least? Could you give an example for me to understand? Also, how do you think the about such integration from the beginning to the end of the programme? Did you find the programme interesting with the social media integration? Could you give an example for me to understand? |
|  | Recall your experiences with the NM programme. Do you think the social media integration has impacted on your reflection and discussion positively? Which aspects do you think it has impacted upon? How? And why? Do you have an example to help me understand? |
|  | How should we improve the NM programme to better facilitate your reflection and discussion positively? |
|  | Do you think that the social media integration creates some barriers or side-effects to your learning? Do you have an example to help me understand? |
| **End Question** | Do you want to share something else about your experiences of the course with the integration of the social media? |
